# Telephones

**Published:** 1901-12-14

**Authors:** 


### The Hospital

A Journal of

The Medical Sciences and Hospital Administration.

Vol. XXXI.—No. 794.] Edited by Sir Henry Burdett, K.C.B., and Solomon C. Smith, M.D., M.R.C.P. [December 14, 1901.

### TELEPHONES.

In prospect of the approaching invasion of the quiet of the county of London by a system of universal telephones, it may not be altogether superfluous to inquire precisely what it is that they will accomplish for the benefit of the inhabitants, and what evils, if any, may be anticipated from their employment. Let us apply ourselves to what an American might perhaps describe as the "bottom fact" of the case; the fact that the great majority of the dwelling-houses of the county will before long be furnished with appliances by means of which the occupants may be disturbed at any moment of their lives for the purpose of replying to the possibly frivolous observations or inquiries of some person who, while in possession only of the previously established methods of communication, would have been constrained to leave them alone. We once knew a distinguished physician who was accustomed to describe the effects upon himself of a bad night by saying that it caused him to forget, until he was half-way downstairs, something which he ought to have said in the sick-room; and the French have the delicious phrase, *l'esprit d'escalier*, to express the conception of a *bon mot* which has flashed upon the mind a moment too late for utterance. There seems to be some risk that life will henceforth be given over to continual resurrections, so to speak, of the particular form of *esprit* in question. The telephone will be the natural channel of a perpetual "by-the-bye" from the bore who has just departed, and from whom we fondly hoped that our deliverance, even if only temporary, was for the time complete. Elia somewhere waxes pathetic over the misery of being interrupted, and his complaint will have tenfold applicability to the wire-borne visitor. The occupant who still regards his house as his castle, and who resolutely refuses to admit the intruder, will certainly, for a time at least, be an exceptional character. His womankind will break into open rebellion. They will tell him that Mr. Jones across the way has the telephone, and that only last week, when visitors arrived at the house unexpectedly, Mrs. Jones was able to speak at once to a London fishmonger, and to have a turbot sent down in time for dinner. Mrs. Robinson, again, when her little Tommy appeared to be choking, called hastily over the wire to the doctor and asked him what to do. He told her to pat Tommy on the back, and the dear child instantly recovered.

The providential preservation of Tommy, effected by the agency above described, points, we fear, to the direction in which telephones threaten to be greater nuisances than in all others put together. A new terror will be added to the lives of the unfortunate members of our calling. Doctors will have no peace, and no ordinary foresight will suffice to protect them. We have heard of a case in which a very eminent surgical consultant was called in to see the youthful heir to an old title and an illustrious house. As he and the family doctor left the sick room together, they were closely followed by the venerable grandmother of the patient, her features expressive of a stern determination to "have it out" with the doctors. The consultant was equal to the occasion. As the door closed behind grandmamma, he wheeled round, and said, in his most bland accents, "I think the little fellow is calling you, my lady." She darted back, and, while she was saying "What is it, my darling?" the consultant, on his part, had said to his colleague, "Goodbye, my dear fellow, self-preservation is the first law of nature," and had actually run down the stairs and reached his carriage in safety, before the character of the alarm which he had given had been discovered. We will drop a veil over the fate which would have awaited him if telephonic connection with his house had been established. For the future, there will be no end to the importunate inquiries of patients. Has medicine been prescribed to be taken with meals, the bell will ring to ask whether it should accompany the first mouthful or the last? At first sight, it would seem possible to arrange a protective tariff of charges for replies by telephone; but the practical difficulties in the way would be serious. We knew a hospital surgeon who received students as boarders, and whose servant complained of having to sit up on account of their late hours. The master met the difficulty by telling the students that his doors were open until midnight, but that if they were out after twelve had struck they must each pay the man a shilling for sitting up to let them in. The man soon complained that the shillings were readily promised, but seldom paid, and he was then authorised to refuse to open the door until the necessary coins had been dropped into the letter-box. Now it seems possible that some sufficiently discreet persons, say, for example, the direct representatives of the profession in the General Medical Council, if, as Sidney Smith said of another matter, "they were to lay their heads together," might arrive at some suitable scale of fees for telephonic communications; but the difficulty of procuring payment might remain. Electricians would have to be called in, in order to devise some contrivance, some "guinea-in-the-slot" machinery, which should fulfil the purpose of the surgeon's letter-box. Of course, as time goes on, compensations for telephonic annoyances will come to light; and a generation will grow up to the members of which they may possibly appear trivial. As far as the present one is concerned, we are in agreement with the *Times* in thinking that the great majority of the users will find telephones not to be unmixed blessings after all.

